# Spatially modulated structural colour in bird feathers

**DOI:** 10.1038/srep18317

**Published:** 2015-12-21

**Authors:** Andrew J. Parnell, Adam L. Washington, Oleksandr O. Mykhaylyk, Christopher J. Hill, Antonino Bianco, Stephanie L. Burg, Andrew J. C. Dennison, Mary Snape, Ashley J. Cadby, Andrew Smith, Sylvain Prevost, David M. Whittaker, Richard A. L. Jones, J. Patrick. A. Fairclough, Andrew R. Parker

**Affiliations:** 1 Department of Physics and Astronomy, The University of Sheffield, S3 7RH, UK; 2 Department of Mechanical Engineering, The University of Sheffield, S3 7HQ, UK; 3 Department of Chemistry, The University of Sheffield, S3 7HF, UK; 4 Department of Molecular Biology and Biotechnology, The University of Sheffield, S10 2TN; 5 University Grenoble-Alpes, IBS, 38044, France; 6 Institut Laue-Langevin, 38042 Grenoble Cedex 9, France; 7 Beamline I22 Diamond Light Source, Oxfordshire, UK; 8 ID02 Beamline, European Synchrotron Radiation Facility (ESRF), F38043, Grenoble, France; 9 Department of Zoology, Natural History Museum, London SW7 5BD, UK

## Abstract

Eurasian Jay (*Garrulus glandarius*) feathers display periodic variations in the reflected colour from white through light blue, dark blue and black. We find the structures responsible for the colour are continuous in their size and spatially controlled by the degree of spinodal phase separation in the corresponding region of the feather barb. Blue structures have a well-defined broadband ultra-violet (UV) to blue wavelength distribution; the corresponding nanostructure has characteristic spinodal morphology with a lengthscale of order 150 nm. White regions have a larger 200 nm nanostructure, consistent with a spinodal process that has coarsened further, yielding broader wavelength white reflectance. Our analysis shows that nanostructure in single bird feather barbs can be varied continuously by controlling the time the keratin network is allowed to phase separate before mobility in the system is arrested. Dynamic scaling analysis of the single barb scattering data implies that the phase separation arrest mechanism is rapid and also distinct from the spinodal phase separation mechanism i.e. it is not gelation or intermolecular re-association. Any growing lengthscale using this spinodal phase separation approach must first traverse the UV and blue wavelength regions, growing the structure by coarsening, resulting in a broad distribution of domain sizes.

The vibrancy and variety of structural colours found in nature has long been well-known; what has only recently been discovered is the sophistication of the physics that underlies these effects^1^,^2^,^3^,^4^,^5^. Bird feathers have proved particularly important in our understanding of structural colour. Structurally coloured feathers are a stable nanostructured material made from β-keratin that can be studied in detail even after collection, although pigmented sections or layers will degrade over time, importantly any nanostructure will remain. Iridescent feathers such as the tail feather of the male Peacock are a vibrant example of the wealth of possible colours. Robert Hooke, a founding father of optical microscopy was one of the first to examine the Peacock^6^,^7^ and Duck^8^ feathers in his revolutionary text Micrographia. He saw that by exposing them to water he could alter the intensity of the colour^9^. Clyde Mason performed a comprehensive study of this effect for the case of the Blue Jay (*Cyanocitta Cristata*). He was able to optically contrast match the permeating solvent to that of the blue feather barbs using Canada balsam (*n* 1.54)^10^. Solvents above and below the refractive index of β-keratin (*n* 1.54)^11^ showed distinct colour, pale blue in the case of the solvent Carbon disulphide (*n* 1.627) and a sea green colour for water (*n* 1.33). The conclusion of this repeated solvent exposure is that the blue colour is not due to a pigment, as this would not explain this switching off and on of the colour.

In this paper we primarily focus on a comprehensive study of the Eurasian Jay and the origins of its structural colour. We also detail spatially modulated structures in a number of geographically diverse birds spanning the globe, from the two dominant types of isotropic structural colours found in nature. These structure formation routes are categorized as sphere forming (nucleation and growth) and channel type (spinodal decomposition). Initially we examine the Eurasian Jay, shown in Fig. 1b, with its distinctive flash coloration on the wing feathers. This pattern is the same for both male and female. It is periodic on the macroscopic scale (Fig. 1a) and along an individual feather barb (see Fig. 6a). The purpose of these markings is still unclear but possible explanations include species recognition at a distance or as a sexual selection signal^12^ where the ultra violet component of the signal could also play a role^13^. When the feather is seen in cross section (Fig. 1c) it is evident that only a thin layer (~10 μm) is needed to provide the effect. The microstructure of individual barbs shows a network of polygonal cells (Fig. 1d,e), responsible for the structural colour, having an appearance of a thick layer of blue enamel, termed “émail” by Fatio^14^,^15^,^16^.

Two-dimensional Fourier analysis of electron microscopy images^17^ showed that blue structural coloration in feathers is due to constructive interference between light waves, coherently scattered by a nanostructured keratin–air matrix. This dispelled the longstanding consensus that blue colour was the result of incoherent Tyndall scattering, agreeing with the conclusion put forward by Raman in the 1930’s^18^. Blue structurally coloured feathers are in effect Bragg reflectors^5^,^19^. Transmission electron microscopy (TEM) images established that the Jay feather’s blue colour is caused by a structure that has the “character of a foam”^20^. TEM observations of sponge-like structures in other bird species (*Cotinga maynana* and *Agapornis roseicollis*) lead to the proposal of the “hollow cylinder model”^21^. In which the sponge like structure consists of randomly oriented hollow cylinders with diameters of around 100 nm.

Looking back in the scientific literature the proliferation of structural blues in nature had always been explained as a direct consequence of its origin as Tyndall scattering, and this was a widely held tenet in understanding this effect. The earliest observations of non-iridescent green feathers nearly always (except for the pigment Turacoverdin found in Turacos) consist of a pigment in combination with a structural blue^10^,^16^,^22^,^23^,^24^. Krukenberg was the first to record this as far back as 1882, however he states “this cannot always be the case”^16^. Over 130 years has passed since this comment and we have found very few examples of non-iridescent structural green, one comes very close^25^, however it still requires dispersion and absorption at short wavelengths. The field at large did not take up this search, as the prevailing view was that the origin of the structural colour was due to Tyndall scattering. The question now finally arises after the discovery by Prum^17^ of its true origin, if Tyndall scattering is not the cause of this optical effect then why do we not see a multitude of structural colours without the need for added pigment layers? The isotropic photonic structures found in bird feather barbs come in two distinct morphologies, these are spherical (nucleation and growth) and channel (spinodal)^5^. Recent measurements on the feather barbs of *Cotinga maynana* bird, a sphere type structure, demonstrated that these are limited to wide reflection spectra due to double scattering of light in these structures^26^. Even though these are able to produce very narrow primary reflection peaks, the secondary reflection peak will always be at a lower wavelength and so broadens the total effective reflectance. This means that isotropic sphere structures are limited in their reflectance colour “pure green or red colours cannot be produced structurally”^27^. The case for spongy spinodal structures will be highlighted later.

## Results

**Figure 2 f2:**
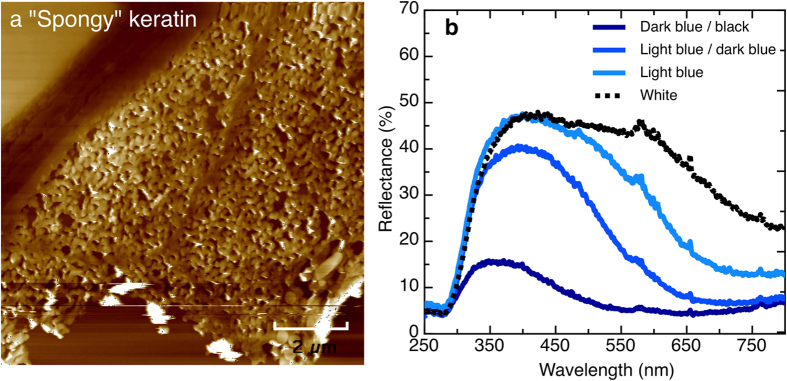
Scanning probe imaging of the dark blue region, showing the “sponge” morphology responsible for the optical properties. (**b**) The measured reflectance for the different regions of a Jay feather covert.

Figure 2a is a scanning probe image of a dark blue region of the Jay feather, clearly showing the sponge morphology responsible for the colour. The long-range ordering of the cylinders is not critical to the creation of the overall structural colour, as light scattered by separate regions of the barb will not efficiently interfere. This lack of long-range periodicity in the spatial correlations explains why very little iridescence is observed in these bird species. As such they have similar colour (spectral) appearance when viewed from various angles. The reflectance spectra (Fig. 2b) for the different regions of the Jay feather barb all have a sharp rise at 290 nm (near UV-A 320 nm–400 nm) and span into the blue wavelength region (visible to human vision). Birds possess tetrachromic vision and have been shown to communicate using these wavelengths^12^,^13^. The peak in the reflectance spectra broadens in the transition from light blue to dark blue and eventually the reflection becomes white in colour. The Raman spectra (Fig. SI 1) provide a chemical map showing that the feathers are predominantly made of β-keratin, however for the darker regions there is some additional melanin^28^, which absorbs the transmitted light.

**Figure 3 f3:**
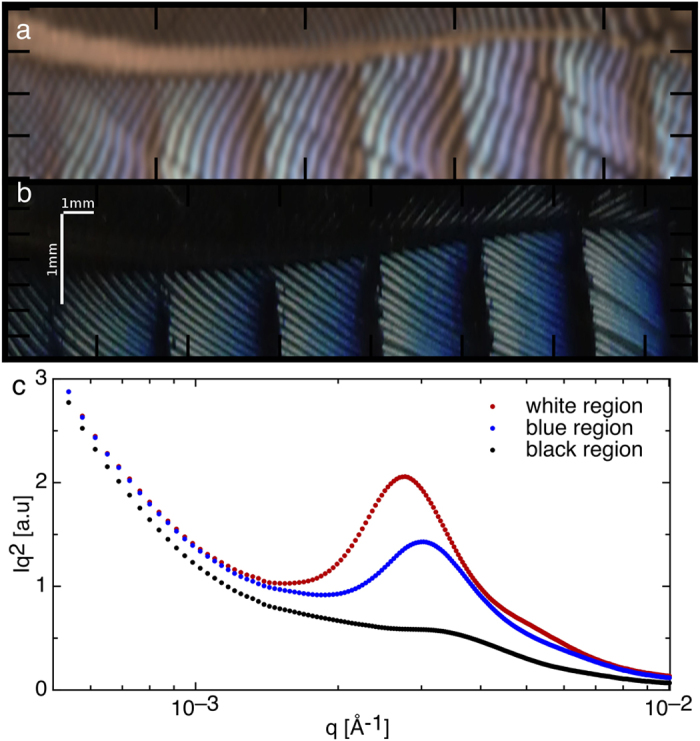
(**a**) The colour of the Jay feather reconstructed from the Small Angle X-ray Scattering (SAXS) scan of the region in (**b**). (**b**) The same region of the feather observed by optical microscopy. In images a and b the horizontal axis spans 22 mm and the vertical axis 2 mm, a scale bar is included in (**b**) part a has the same scale in the x and y directions (The wavy periodic lines in Fig. 3a. are a moiré pattern due to the superposition of the SAXS beam sampling position and the barb separation. When the grid pattern used for the SAXS scan is imposed on the optical image, the same effect is observed, see Fig. SI 2) (**c**) Representative Lorentz-corrected SAXS data for the different coloured regions of a Jay feather barb (measured at ID2 (ESRF)).

To probe the lengthscales present in the Jay feather we have used small angle X-ray scattering (SAXS), which is a proven technique used to characterize the lengthscalesles present in a number of structurally coloured bird feathers^29^. Importantly no sample preparation is required, unlike a typical electron microscopy specimen, and the structure is therefore unperturbed. The colour map for the Jay feather in Fig. 3a was reconstructed using the optical wavelength spectrum derived from the Fourier transform of the corresponding SAXS data^17^, which in Fourier space gives the contributions that provide the total optical reflectance spectra. This data shows a periodic modulation of the domain size and consequently the structural colour as a function of position, which to date has not been seen in these quasi-ordered nanostructures. The colour in Fig. 3a extracted from the SAXS data faithfully matches the observed colour in the optical image (Fig. 3b), showing the link between nanostructure and optical properties. Representative SAXS data for the different colours are plotted in Fig. 3c for the white, blue and black regions (Fig. 3c). Given the q-range available to us in our SAXS setup we were able to follow the large dynamic range in structure that the feather barbs span.

To date a handful of studies have examined structural whites^3^,^30^. One of the few studies to look at birds examined the Rock Ptarmigan (*Lagopus muta*), and found that an air β-keratin network is responsible for the white colour^30^, producing ~50% broadband spectral reflectance across the visible wavelengths from a single feather, similar to that of the white region in the Jay feather.

**Figure 4 f4:**
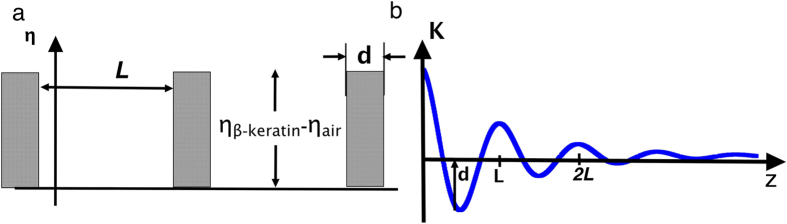
(**a**) A model two phase system with discrete values of the electron density η_β-keratin_ and η_air_ for the two phases, the dark regions are the β-keratin domains in our system and the second phase air is the lower value. The long period is the distance between the midpoints of each domain, whilst the β-keratin domain width is simply the width d. In b we can see how these two parameters are reflected in the real space correlation function.

In order to fully interpret the SAXS data and extract the real space morphology and transitions in size we have used one dimensional correlation analysis. An inversion of this type has been performed previously on a scattering dataset for the plum throated Cotinga (*Cotinga Maynana)*, a sphere forming structure^26^.This approach has also been used extensively in the field of semi-crystalline polymers, using the software known as CORFUNC to look at amorphous crystalline lamellae^31^. Using only the assumption that the sample is a two-phase system with differences in electron density, the auto-correlation function allows for the extraction of several properties of the underlying structure, such as the long period or the domain width of the phases. Figure 4 shows the model and the relevant parameters, long period and domain width. The process involves fitting the low-q scattering data to a Guinier curve and the high-q data to a Porod curve, and then it is possible to create an extrapolation of the small angle x-ray signal that extends over all q. This extended function can then be Fourier transformed to return the auto-correlation of the electron density within the sample.

**Figure 5 f5:**
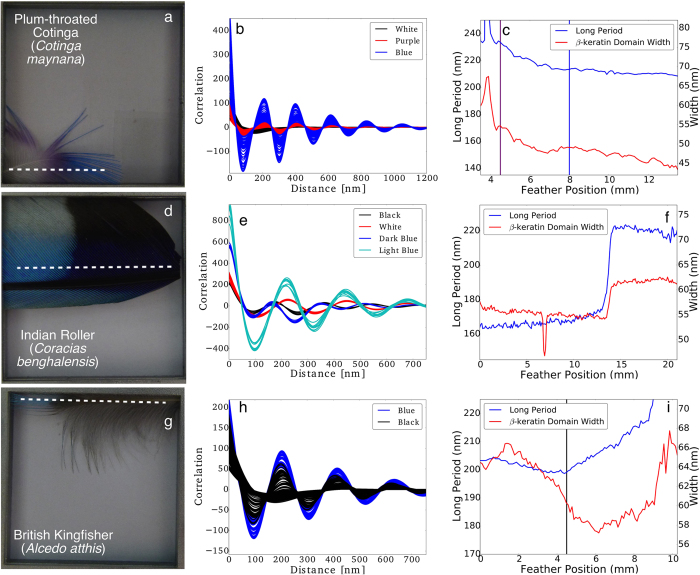
One dimensional correlation function analysis of SAXS scans of structural colour transitions for three further bird species. (**a**) The Plum-throated Cotinga (*Cotinga maynana*), (**d**) the Indian Roller (*Coracias benghalensis*) and (**g**) the British Kingfisher (*Alcedo atthis*) along with the correlation functions (**b,e** and **h**) using one dimensional correlation analysis, the y scale in (**b,e** and **h**) is arbitrary, it is merely the extent of the correlation length that is important. The derived long period and β-keratin domain width (**c**,**f** and **i**).

We have also found similar structural colour transitions like those found in the Jay for a number of geographically diverse avian feathers, including the Plum-throated Cotinga (*Cotinga maynana*), British Kingfisher (*Alcedo atthis*) and the Indian Roller (*Coracias benghalensis*). The transitions for these three bird species are displayed in Fig. 5.

The Cotinga shown in Fig. 5, has already been categorized as a sphere forming structure^29^. As such it has much higher local order when compared to the spinodal structures of the British Kingfisher and the Indian Roller. We can see this clearly in the difference between the correlation function in Fig. 5b, which extends much further out in distance (~1200 nm) than the Indian Roller in Fig. 5e (~700 nm) or the British Kingfisher in Fig. 5h (~700 nm). The transition for the Cotinga from purple to blue is highlighted by the white dotted line in Fig. 5a and the extracted parameters from the one dimensional correlation analysis are shown in Fig. 5c. Going from left to right the long period falls below 220 nm as it transitions from purple to blue, the domain width also reduces towards 140 nm in the blue region. The Indian Roller shows a sharp transition zone from light blue on the left to dark blue with a jump in the long period from 160 nm to 220 nm and an increase in the β-keratin domain width of 5 nm. The British Kingfisher in Fig. 5g transitions from blue to black and has an initial drop in the domain width followed by a gradual increase, the long period gradually increases after the transition point. The fact that we find similar colour transitions in a diverse array of birds from different regions of the globe shows that control over barb photonic structure is a general phenomenon.

**Figure 6:**
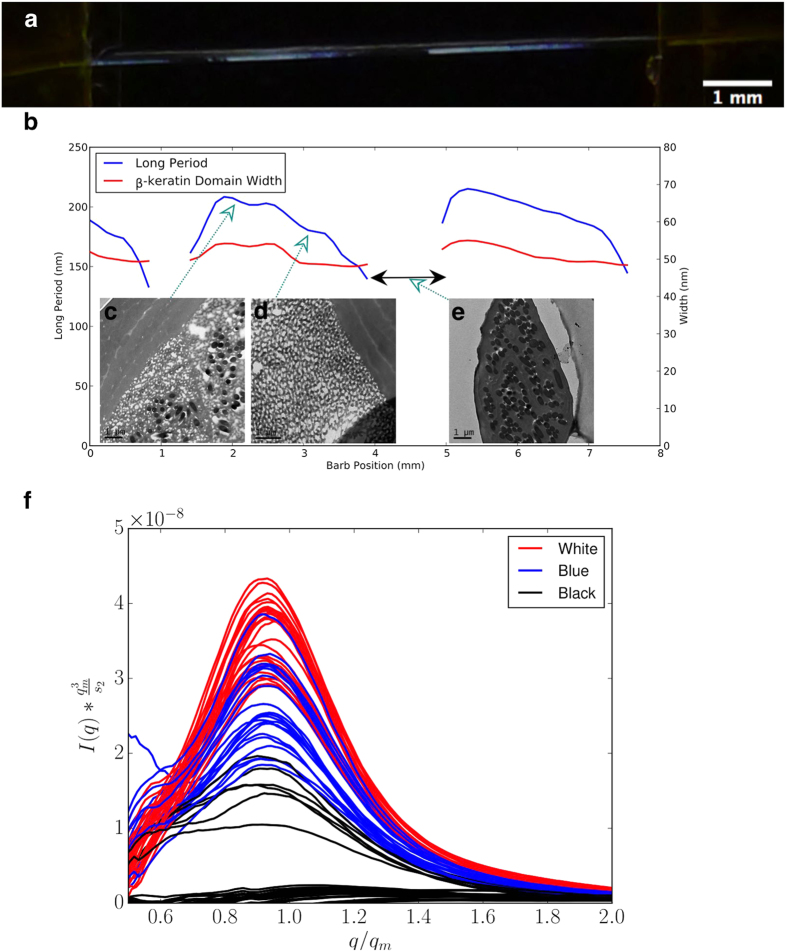
(**a**) Periodic variation in the Jay feather structure and colour across a single feather barb. (**b**) The long period, which is the spacing between neighbouring β-keratin air domains, and the β-keratin domain width. The SAXS scattering was measured as a function of position across the single Jay feather barb, and used one dimensional correlation analysis^31^ of the SAXS data. Electron microscopy (EM) images for the different coloured regions of the jay feather; (**c**) white region, (**d**) dark blue region and (**e**) the black region, where melanin creates the strong absorption of light and no clear β-keratin structure is observed in SAXS nor EM. (**f**) dynamic scaling of the scattering data for the different regions of the feather barb.

To further understand the structural colour and phase separation mechanism in the Jay we have performed a SAXS scan of a single feather barb (Fig. 6a), which represents the subunit of a feather. The same periodic pattern in oscillatory nanostructure is seen in the feather barb, using one dimensional correlation analysis we extract the peak length present at each point along the feather, clearly showing an oscillating pattern (Fig. 6b). This validates our assessment that the oscillation in nanostructure is real and not due to the sampling of a number of barbs, as we have unambiguously mapped the structure for a single barb. This points to a greater complexity in the mechanisms and nanoscale ordering present in the developing Jay feather^10^, allowing the optically active nanostructure to be controlled according to the position along the barb during growth. To reiterate, the lengthscale of the optically active nanostructure is not discrete and quantized; it has strong variation and tunability, based on the position along the feather barb. The real space images for white, blue and black (Fig. 6c–e) are shown for the respective regions showing the change in lengthscale as a function of position along the feather (further TEM images are displayed in Figure SI 4).

## Discussion

Previous work has postulated that the spongy keratin structures responsible for structural colour in feathers arise via the physical process of spinodal decomposition^32^,^33^. A TEM study examining the early growth stages of feather barbs in the blue and yellow macaw (*Ara ararauna*)^33^ proposed intracellular spinodal phase separation of the polymerizing β-keratin from the rest of the cytoplasm, as the mechanism behind nanostructure formation. However in both these studies it was a hypothesis without evidence to prove the mechanism, they concluded that the β-keratin morphologies looked like spinodal patterns based on electron microscopy images and one scattering curve. In order to prove this conclusively an analysis of the developing system is needed as a function of time. In our study of the Jay we examine single barb and so are able to compare a series of different effective phase separation timescales, albeit not from the same position.

Spinodal decomposition^32^,^34^ is a process by which random fluctuations in composition of a liquid mixture are selectively amplified, resulting in a pattern which is spatially random but characterized by an inherent lengthscalele, giving a scattering pattern with a characteristic peak, as seen in figure Fig. 3c. At a later stage, the phase separation pattern coarsens, creating larger domains to minimize total interfacial energy^34^,^35^. The increase in lengthscale can be seen in reciprocal space by the shift in the peak position q_m_ to smaller wave vectors (Figure SI 5), in real space this is an increase in the long period lengthscale shown clearly in Fig. 6b as a function of position along the barb.

For spinodal decomposition to lead to a permanent nanostructure of the kind we see in the β-keratin foams in bird feather barbs, there must also be some additional control mechanism to arrest the phase separation and lock the structure in place. Otherwise the structure would simply phase separate and it would not be possible to have the spatially modulated photonic structures that we have directly measured using SAXS in this work. The entire barb would have to reach some end point and be all one colour. Our work suggests that in the Jay feather, it is by controlling the duration of the phase separation process before this arrest takes place, which provides modulation and fidelity over the feather colour. In the white region of the barb, coarsening of the β-keratin foam structure proceeds to a greater extent than in the blue region, before the phase separation is arrested. The ability to arrest the phase separation at any point in the coarsening process is what leads to the continuous tuning of colour.

Potential mechanisms for arresting phase separation can be physical, for example, crystallization or other physical association mechanisms in the macromolecule-rich phase, or chemical, through the formation of a cross-linked network^36^. Currently we are unable to identify the biological mechanism at work here, but further analysis does give a clue as to its character. In late stage spinodal decomposition, theory shows simple systems are characterized by the remarkable property of self-similarity. As coarsening proceeds, the evolving structures are self-similar – that is, a pattern at later time is, on a statistical basis, simply a magnification of the pattern at an earlier time. This self-similarity is manifested most clearly as a simple scaling property of the scattering curves. Figure 6f shows the results of this dynamic scaling analysis applied to the single barb SAXS scattering data^37^,^38^,^39^. The failure of the curves to totally superpose indicates that strict self-similarity does not apply. But a comparison of this case with another study of phase separation in a biopolymer system^40^ suggests that the departure from self-similarity is much weaker than in situations when the arrest of phase separation is effected by the simultaneous and slow process of intermolecular association. This suggests that the phase separation arrest mechanism occurs over a time-scale that is widely separated from the time-scale characterizing the phase separation and coarsening, implying a two-stage process. This contrasts strongly with the prevailing school of thought that the phase separation process is halted by the competition between phase separation and the polymerization of β-keratin, which gives no control over the final lengthscale^33^.

Spongy-type structural colour reflectors are versatile in their range and flexibility, from the UV through the visible, with tremendous variation in the possible colours when combined with pigments^41^. The explanation for why blue producing spinodal structures are able to make a blue structural colour using a broad size distribution is that humans do not see wavelengths in the UV. As spinodal structures may exhibit a spread in lengthscales, and therefore significant short wavelength reflectance in the UV spectrum, without affecting the reflected blue colour. As opposed to previous works, the data we present comprise the full range of spinodal lengthscales without gap, proving conclusively that we have shown that the structural colour from this spinodal structure is constrained to UV, blue and white, and so requires the use of pigments for the full colour range.

**Figure 7:**
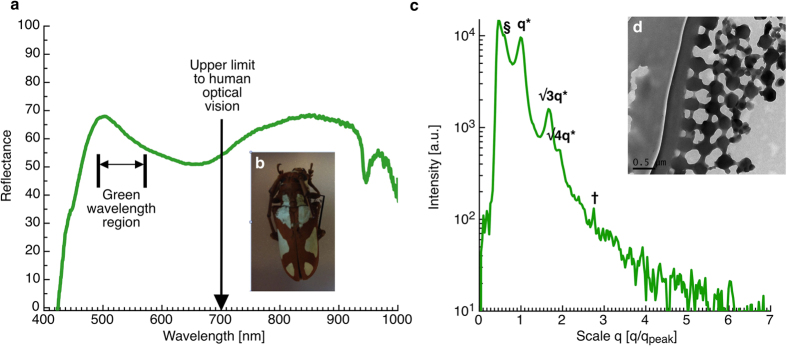
Reflectance measurements for the green region of the elytra of the African Longhorn Beetle (*Prosopocera lactator*). An image of this beetle is shown in (**b**) clearly showing the green regions of its elytra. (**c**) small angle x-ray scattering data taken from the green region of the African Longhorn Beetle elytra. The structure is scaled and indexed according to the peak at q* (1.83 × 10^−2^ nm^−1^) 343 nm and √3q* and √4q*. Other peaks can also be seen at q values of § (1.12 × 10^−2^ nm^−1^) = 560 nm and † (5.03 × 10^−2^ nm^−1^) = 124.8 nm. (**d**) a TEM image of the green region of the African Longhorn Beetle elytra.

This use of pigments to augment the spinodal and sphere type structural colours is common in the Natural world. In particular yellow carotenoid pigments are common in bird feathers, however green pigments are particularly rare in animals; as such other architectures for producing structural greens may be too complex to evolve in feathers (particularly if iridescence is not selected for). An example of one of the very few known non-angular dependent structural greens is made using a true photonic structure that is found in the elytra of the African Longhorn Beetle (*Prosopocera lactator* see Fig. 7). To circumvent the iridescence that this photonic structure would normally produce, the elytra is made up of small locally ordered domains that are arranged in different orientations to disrupt iridescence^25^. The green reflectance of this particular beetle however still relies on significant absorption and optical dispersion at short wavelengths due to Chitin^25^. This particular beetle structure clearly shows how precise and well defined the nanostructure must be to produce a true structural green. This may well be the minimal design to perform this optical function.

## Conclusions

By directly measuring the nanostructure of a Jay feather, we show for the first time that the variation in color along a single feather barb is achieved by continuous modulation of its nanostructure, leading to variation of the structural color. These nanostructures are produced by a phase-separation process that is arrested at a late stage; mastery of the color is achieved by control over the duration of the phase-separation process. This phase separation mechanism is likely to be widely shared amongst birds, reptiles and amphibians. For example the green tree frog (*Litoria caerulea)* reverts to a blue colour after death, due to degradation of the yellow pigmentary material^42^. The observation that tuneable broad wavelength spinodal structures are constrained in their choice of colours explains why non-iridescent green has to be made using a mixture of a structural blue colour and a yellow pigment^24^, and finally answers the longstanding conundrum of why green, an obvious camouflage colour, is not prevalent in the natural world as solely a structural colour^10^,^16^,^22^.

## Methods

Reflectance -For the optical reflectance measurements an Ocean Optics USB2000+ spectrometer was used with a UV transmissive fibre optic cable. This fibre has a *y* shaped arrangement (3 ended –with one probe fibre (dual input/output) onto the sample and the other two connections into the light-source and spectrometer respectively). A DT-MINI-2-GS (Ocean Optics) Deuterium Tungsten Halogen UV-Vis-NIR light source was used, which has a wavelength range from 215 nm–2500 nm. The illumination was placed directly onto the feather sample at 90° to produce a small spot of light ~1.0 mm in diameter.

The reflection spectrum was measured for the different coloured regions of the feather, with the electrical dark current subtracted (a shielding was placed over the spectrometer and the signal was recorded with no light input). For all the measurements three scans were averaged and the boxcar width of 3 nm was used. The positional feather reflectance data for the intensity as a function of wavelength was referenced to the reflectance from a white reference standard (WS-1-SL Spectralon reflectance standard 99% 400 nm–1500 nm and >96% 250−2000 nm, Ocean Optics).

Optical microscopy. The optical images were collected using a Nikon ME600 optical microscope mounted on an isolation table and fitted with a Pixelink PL-A742 machine vision camera, and using a 5×, 10×, 20×, 50× or 100× magnification objective (Nikon). A calibration graticule was used to calibrate the lengthscale for the optical images. An analyser and polarizer were placed in the optical train, and the polarizer rotated to enhance the contrast. The program ImageJ was used to add a scale bar to the optical images.

Transmission electron microscopy. The Jay feather sample was dehydrated in 100% ethanol over several days; the ethanol was replaced with 1,2 Epoxypropane (EPP), the EPP was exchanged twice, each after 1 hour. The EPP was replaced with a 1:1 mixture of EPP:Araldite resin. The feather was left in the1:1 solution mixture overnight, on a rotating mixer. The solvent was then replaced with Araldite resin for 6–8 hours (with two changes of 4 hours each, again agitated on a rotating mixer). Finally the solvent was replaced with fresh Araldite CY212 resin (Agar Scientific) for embedding in “coffin moulds” before curing for a period of 48–72 hours at 60 °C. Semi-thin sections of approximately 0.5 μm thickness were cut on an ultra-microtome (Reichert-Jung Ultracut E) and stained with 1% toluidine blue in 1% borax for around 30 seconds on a hotplate or until the stain began to evaporate. The sections were washed in water, dried on a hotplate and mounted in DPX (Agar Scientific) with a cover glass. Ultrathin sections, approximately 85 nm thick, were cut on an ultramicrotome (Reichert-Jung Ultracut E) on 200 mesh copper grids (Agar Scientific). The feather sections were examined using a Transmission Electron Microscope (FEI Tecnai) at an accelerating voltage of 80 kV.

Raman spectroscopy. The Raman microscopy measurements were taken using a Renishaw inVia system (Renishaw UK). The Jay feather sample was focused via the white light system and then fine adjusted to maximize the signal counts into focus using the Argon ion laser (514.5 nm) illumination.

Scanning probe microscopy

The feather barbs were characterized using scanning force microscope (Veeco Dimension 3100) operated in tapping mode. The AFM tapping mode tips were Olympus with a resonance near 275 kHz. The AFM images were analysed and images produced using the program ImageSXM.

The initial Small Angle X-ray (SAXS) experiments measurements of specific regions on the Jay feather were performed at Spring8 (Japan) using the beamline BL03XU, followed by subsequent measurements on the Jay feather at the Diamond Light Source (UK) using the beamline I22. Finally feather maps and a scan of a single feather barb were done on ID2 at the ESRF (Grenoble, France). At Diamond a Pilatus P3-2M 2D detector positioned at a distance of 9.575 m from the sample was used with an X-ray wavelength of 1.2 Å (10 keV). A letterbox shaped beam profile was used (400 μm horizontal × 200 μm vertical). This setup gave an upper resolvable lengthscale of 620 nm. Whilst at the ESRF a Rayonix MX-170HS detector was used with 1 Å wavelength X-rays, a beam size of 20 μm by 20 μm and a sample-to-detector distance of 15 m or 30 m. For the single Jay feather barb measurement a rotation stage (Newport) was used to align the feather barb relative to the X-ray beam and so enable a linear scan of the barb. The feather map SAXS data was Fourier transformed, and the resulting wavelength spectrum converted into x,y and z (CIE 1931 colour space values) and then subsequently converted into RGB values. The SAXS barb data was analysed using the software CORFUNC^31^, implemented in Python. This program was used to determine the correlation function for each measured region of the barb nanostructure, based on the integrated two-dimensional SAXS data. The program analyses the one-dimensional SAXS using correlation function analysis in terms of an ideal lamellar morphology. Crucially it generates a model independent volume fraction profile from a one-dimensional SAXS pattern, with no free fitting parameters. The long period is the distance between the midpoints of neighbouring β-keratin domains. The dynamic scaling analysis was performed on the lorentz corrected barb SAXS data. Firstly by subtracting the background, using a polynomial fitting approach. Subsequently fitting the peak lengthscale (q_m_) in I(q) and then multiplying the scattering data by q_m_^3^/s_2_. Where s_2_ is the total scattering, often called the invariant.

## Additional Information

**How to cite this article**: Parnell, A. J. *et al.* Spatially modulated structural colour in bird feathers. *Sci. Rep.* **5**, 18317; doi: 10.1038/srep18317 (2015).

## Supplementary Material

Supplementary Information

## Figures and Tables

**Figure 1:**
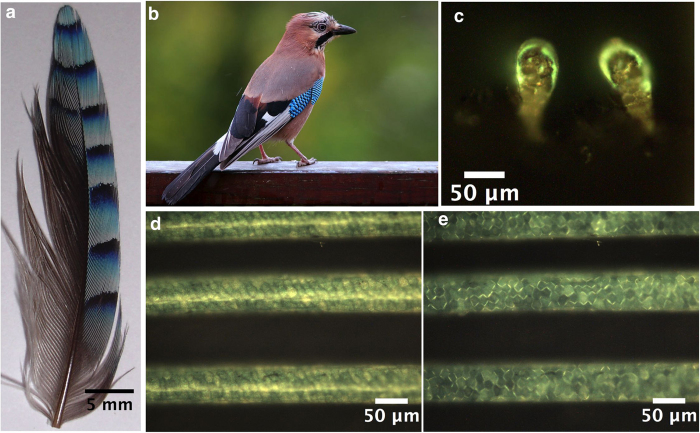
Optical images of a Eurasian Jay (*Garrulus glandarius*) feather at different lengthscales. (**a**) The periodically patterned Jay feather covert, with a period of around 4.5 mm. (**b**) Photograph of the Jay *Garrulus glandarius* (Credit: Luc Viatour/www.Lucnix.be) this image is licensed under the Attribution-ShareAlike 3.0 Unported license. The license terms can be found on the following link: https://creativecommons.org/licenses/by-sa/3.0/” (**c**) A transverse cross section of the light blue part of a Jay feather barb, showing the dorsal portion of the barb with a thin protective outer sheath on top of the vividly blue region, which resembles an arc of colour. d and e, optical microscopy images of the light blue region, the boundaries of these cells are ordinarily invisible under reflected light. In e using a polarizer and an analyser in the optical path, it is possible to distinguish the polygonal cells boundaries, which look distinctly like Voronoi tessellation structures. These range in size from 10 μm–20 μm in diameter and similarly in depth. The blue colour in the barbs is located in the polygonal cells^22^ due to a porous network with dimensions smaller than the wavelength of light^23^.
