# Supplementary material for: Spatially modulated structural colour in bird feathers
Source: Sci Rep. 2015 Dec 21;5:18317. doi: 10.1038/srep18317 (PMC4685390; doi:10.1038/srep18317)
Supplement: Supplementary Information [file srep18317-s1.doc]

**Supplemental Information**

**Spatially modulated structural colour in bird feathers**

Andrew J. Parnell, Adam L. Washington, Oleksandr O. Mykhaylyk, Christopher J. Hill, Antonino Bianco, Stephanie L. Burg, Andrew J. C. Dennison, Mary Snape, Ashley J. Cadby, Andrew Smith, Sylvain Prevost, David M. Whittaker, Richard A. L. Jones, J. Patrick. A. Fairclough and Andrew R. Parker,

**
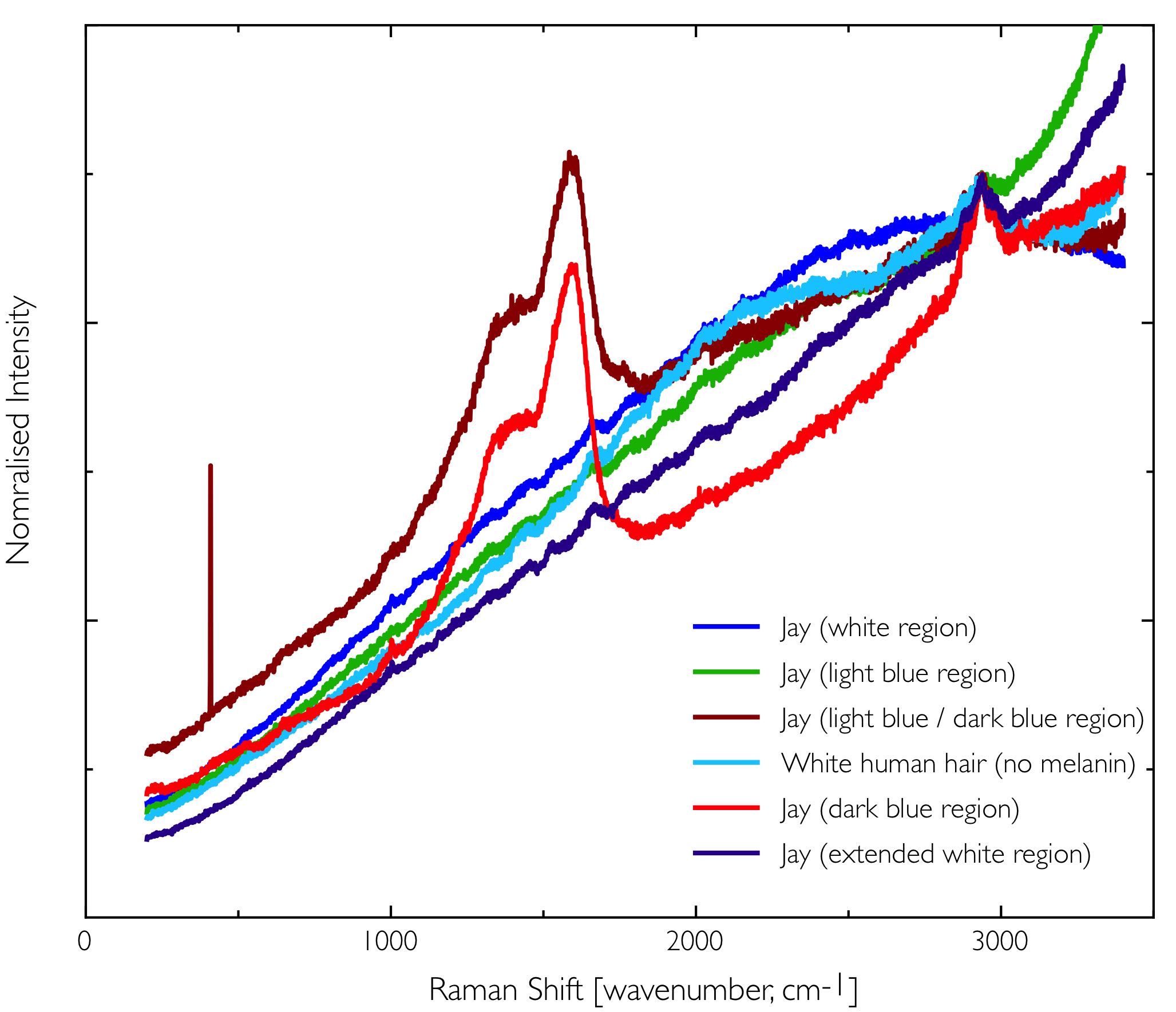
**

**Figure SI 1.** Raman spectra for regions of the Jay feather scaled to the feature at 2935 cm^-1^. This feature is present in all of the Raman data and is a signature of β-keratin. The melanin peak appears at wavenumber 1588cm^-1^ and 1408cm^-1^, and is only present in the darker regions of the Jay feather, these originate from the in-plane stretching of the aromatic rings and linear stretching of the C-C bonds within the rings, and are accompanied by components from the C-H vibrations in both the methyl and methylene groups. Using white human hair, which is melanin free and solely composed of β-keratin as a baseline, we see that the structural colour regions without melanin are composed almost exclusively of β-keratin.

**
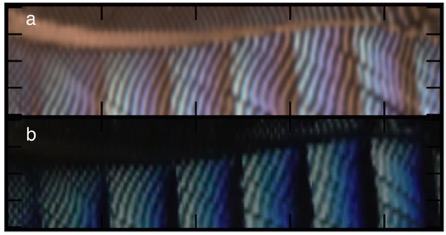
**

**Figure SI 2.** **A,** The line pattern superimposed at an angle of ~ 70 º to the horizontal is a moiré pattern due to the finite size of our X-ray beam and the convolution with the Jay feather barbs. Imposing the same grid used pattern for collection of the small angle x-ray data scan in SI 2 **a**, on the jay feather optical image produces the same pattern in **b** i.e. a moiré pattern, as is seen on the SAXS scan image.


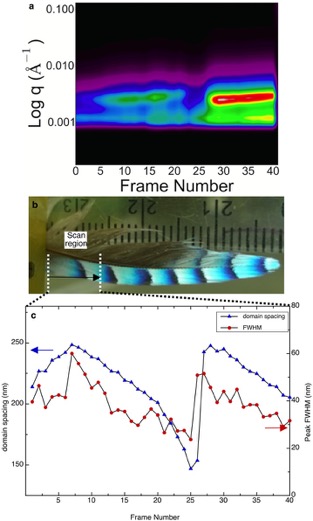


**Figure SI 3.** **a** Small angle X-ray scattering (SAXS) data showing a contour plot for an 8 mm linear scan region, spanning the white region at the left through the blue regions and back into the white region. The data is represented in Lorentz corrected IQ^2^ format in the z direction. **c** Peak position periodic variation in the Jay feather structure and colour (and scan region encompassing the sample between the two white dotted lines in **b**), (from the SAXS data in part **a**). This data was taken on I22 at the Diamond Light Source.

The data in Figure SI 3 was analysed using a much cruder approach than one dimensional correlation analysis and consequently the magnitude of the domain spacing is different, however the periodic change in the domain spacing is clear.


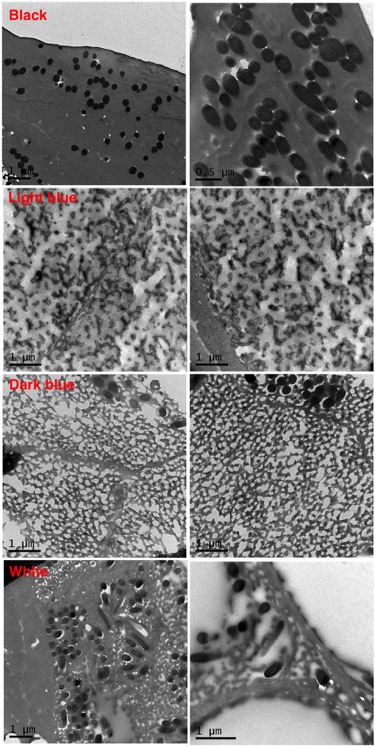


**Figure SI 4.** Further TEM images for the distinct regions of the feather barb, black, light blue, dark blue and white. There are two images for each region and they are paired.

**Figure SI 5.** Peak height values of
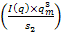
for the various positions on the feather barb in figure 6a (in green on the right y scale) and the corresponding position of the peak lengthscale (blue line) in q space (q_m)_ as a function of barb position.
